# Current State of Knowledge Regarding WHO Critical Priority Pathogens: Mechanisms of Resistance and Proposed Solutions through Candidates Such as Essential Oils

**DOI:** 10.3390/plants11141789

**Published:** 2022-07-06

**Authors:** Bianca Badescu, Valentina Buda, Mirabela Romanescu, Adelina Lombrea, Corina Danciu, Olivia Dalleur, Angele Modupe Dohou, Victor Dumitrascu, Octavian Cretu, Monica Licker, Delia Muntean

**Affiliations:** 1 Doctoral School, “Victor Babeş” University of Medicine and Pharmacy, 2 Eftimie Murgu Street, 300041 Timisoara, Romania; bianca.badescu@medicis.ro; 2 Faculty of Pharmacy, “Victor Babeş” University of Medicine and Pharmacy, 2 Eftimie Murgu Street, 300041 Timisoara, Romania; adelina.lombrea@umft.ro (A.L.); corina.danciu@umft.ro (C.D.); 3 Research Center for Pharmaco-Toxicological Evaluation, “Victor Babes” University of Medicine and Phamacy, 2 Eftimie Murgu Square, 300041 Timisoara, Romania; muntean.delia@umft.ro; 4 Louvain Drug Research Institute, Université Catholique de Louvain, Avenue Emmanuel Mounier 73, 1200 Brussels, Belgium; olivia.dalleur@uclouvain.be (O.D.); angele.dohou@uclouvain.be (A.M.D.); 5 Faculté des Sciences de la Santé, Université d’Abomey Calavi, Cotonou 01 BP 188, Benin; 6 Faculty of Medicine, “Victor Babes” University of Medicine and Pharmacy, 2 Eftimie Murgu Square, 300041 Timisoara, Romania; dumitrascu.victor@umft.ro (V.D.); cretu.octavian@umft.ro (O.C.); licker.monica@umft.ro (M.L.); 7 Multidisciplinary Research Center on Antimicrobial Resistance, “Victor Babeş” University of Medicine and Pharmacy, 2 Eftimie Murgu Street, 300041 Timisoara, Romania

**Keywords:** healthcare associated infections, antimicrobial resistance, MDR strains, *Acinetobacter baumannii*, *Pseudomonas aeruginosa*, *Klebsiella pneumoniae*, *Escherichia coli*

## Abstract

The rise of multidrug-resistant (MDR) pathogens has become a global health threat and an economic burden in providing adequate and effective treatment for many infections. This large-scale concern has emerged mainly due to mishandling of antibiotics (ABs) and has resulted in the rapid expansion of antimicrobial resistance (AMR). Nowadays, there is an urgent need for more potent, non-toxic and effective antimicrobial agents against MDR strains. In this regard, clinicians, pharmacists, microbiologists and the entire scientific community are encouraged to find alternative solutions in treating infectious diseases cause by these strains. In its “10 global issues to track in 2021”, the World Health Organization (WHO) has made fighting drug resistance a priority. It has also issued a list of bacteria that are in urgent need for new ABs. Despite all available resources, researchers are unable to keep the pace of finding novel ABs in the face of emerging MDR strains. Traditional methods are increasingly becoming ineffective, so new approaches need to be considered. In this regard, the general tendency of turning towards natural alternatives has reinforced the interest in essential oils (EOs) as potent antimicrobial agents. Our present article aims to first review the main pathogens classified by WHO as critical in terms of current AMR. The next objective is to summarize the most important and up-to-date aspects of resistance mechanisms to classical antibiotic therapy and to compare them with the latest findings regarding the efficacy of alternative essential oil therapy.

## 1. Introduction

Antimicrobial resistance (AMR) is a process that occurs naturally and has been known for more than 50 years, when *Staphylococcus aureus* began to develop penicillin resistance [1]. Nowadays, AMR has become a public health emergency, mainly due to inappropriate use of antibiotics (ABs) [2]. In addition, socioeconomic aspects such as: poor community hygiene, poor control of hospital infections, use of antibiotics in the animal and food industry are key determinants for the development of AMR [3,4].

In 2017, the World Health Organization (WHO) published a list of bacteria that are in urgent need for new ABs. The following bacteria were classified as having critical priority: *Acinetobacter baumannii*, *Pseudomonas aeruginosa* and *Enterobacteriaceae (Enterobacterales)*. *Enterobacterales* include: *Klebsiella pneumoniae*, *Escherichia coli*, *Enterobacter* spp., *Serratia* spp., *Proteus* spp., *Providencia* spp. and *Morganella* spp. Next on the list are the priority ones: *Enterococcus faecium*, *Staphylococcus aureus*, *Helicobacter pylori*, *Campylobacter* spp., *Salmonellae* and *Neisseria gonorrhoeae* [5].

The reality of AMR in Europe is highlighted by the “Surveillance Atlas of Infectious Diseases”—an ample database conceived by European Centre for Disease Prevention and Control. Situation varies broadly from country to country, but south-eastern region seems to be the most affected [4,6]. For instance, in 2020, Bulgaria was the most affected European country by *K. pneumoniae* resistance to 3rd-generation cephalosporins (Table 1). Even worse, *Acinetobacter* spp. were resistant to carbapenems in most European countries, with Croatia ranking first (Table 2) [6]. These facts are by no means recent. According to previous preliminary findings of a European study, southern and eastern Europe have greater rates of AMR than northern Europe, since these regions consume more ABs per person [7]. Additionally, people in several southern European nations are more fearful of getting sick than those in northern Europe. Other influences are variations in prescription reimbursement programs, the accessibility of over-the-counter ABs, and drug company marketing [8].

A very important factor in increasing global AMR is strongly connected to disruptions in the “one health” principles [9]. The massive and inadequate use of ABs in humans, animals or agriculture has led to the growth in microorganisms resistant to the drugs available today [4,9]. Antibiotic-resistant microorganisms can be transmitted in various ways, such as via water, food or waste [10].

The animal industry represents a key element in the development of AMR. In developed countries, 60–80% of all ABs are purchased for animals, especially poultry, pigs and cattle [11,12]. Moreover, the highest rate of AMR targets the most commonly used ABs in the animal industry: penicillin, tetracyclines and sulfonamides [11]. In addition to ABs, the use of metal ions contributes to the occurrence of AMR. For example, cattle are fed zinc and copper to promote growth and productivity. This elevated AMR of fecal bacteria, since copper-resistant bacteria have shown resistance to ABs such as ampicillin and sulfanilamide [12,13]. Moreover, smaller farms seem to be better protected from antibiotic-resistant microorganisms than larger ones through better management [12,13]. This is due to the superior stringency in cleaning animals or stables, and the effective separation of healthy animals from sick ones [13].

One other important factor involved in AMR is related to the way water is processed [14,15]. A fraction of ABs ingested by humans or animals are subsequently excreted and reach the sewer [14,15]. From there, some ABs pass into natural water sources used in agriculture or for animals. In Italy, a study revealed that 273 *E. coli* strains isolated from water used in agriculture were resistant to ampicillin, tetracycline, sulfamethoxazole and streptomycin [14].

In 2015, the WHO has developed an action plan to combat AMR. Among its objectives, hygiene and sanitation measures were mentioned, as well as reducing the use of antibiotics in human and animal health and increasing investment in new drugs and vaccines, as part of the main strategies to combat the spread of resistant restrains [16].

Since 2017, nine new antibacterial active ingredients were approved by the Food and Drug Administration (delafloxacin, eravacycline, meropenem—vaborbactam, plazomicin, omadacycline, cefiderocol, pretomanid, relebactam—imipenem/cilastatin and lefamulin) and one vaccine for tuberculosis [17]. Nearly half of the recently approved ABs act against carbapenem-resistant *Enterobacterales*, Oxacillinase-48-producing *Enterobacterales* and β-lactamase-producing *Enterobacterales* [17]. A total of 32 antibiotics act on the pathogens mentioned in the WHO’s priority pathogens list [17]. However, new treatment options for carbapenem-resistant *A. baumannii* and carbapenem-resistant *P. aeruginosa* are still lacking [18]. It is worth mentioning that carbapenems are considered as a last line of drugs for the treatment of severe infections. Thus, the increasing frequency of Gram-negative bacteria producing extended spectrum enzymes able to inactivate carbapenems is a major public health concern [19].

Essential oils (EOs) are lipophilic, volatile compounds extracted from various parts of the plants (such as: leaves, roots, stem, flowers, buds, fruits, seeds, and woods), known for their antipathogen properties (such as: antibacterial, antifungal, antiviral, and insecticidal properties) [20]. They are secondary metabolites produced by the plants in order to counter fight the aggression of pests and predators, enhance seed dispersal or to attract the pollinators [20]. They represent a source of robust antimicrobial agent or reversal substances of drug-resistant strains against aggressive microbes [21]. Their wide spectrum of applications encompasses cosmetics (including perfumery and soap or even insect repellents), the beverage and food industry (such as flavoring agents, preservation additives, and disinfecting agents), agriculture (such as pesticides, fungicides or insecticides), as well as the drug industry (potent antimicrobial agents used in controlling healthcare related infections or as an alternative to medico-therapeutic techniques) [22].

Several studies reported EOs which possess direct antibacterial properties, as well as sensitizing or re-sensitizing actions on drug-resistant strains against pathogenic bacteria [23]. EOs are seen as agents with a great antimicrobial potential based on their natural origin. The high antibacterial effect of EOs was observed especially in members of *Lamiaceae* family and was attributed to the high content of thymol and carvacrol (monoterpenoid phenols, derivates of cymene) [24], even if, in some genera, the composition of essential oils can change, even considerably, depending on the different environmental conditions [25,26]. Data regarding the bioavailability of EOs in humans are extremely limited, with oral, pulmonary and dermal absorption being considered effective [20]. Due to their lipophilic structure, EOs can easily cross cell membranes, including the blood–brain barrier (with psychological effects in several central nervous system (CNS) diseases such as: anxiety, depression, insomnia, etc.) [27]. A dose-dependent selectivity of EOs was noticed, as well as a non-toxic effect against normal human cells has been demonstrated, although for the moment, limited data are available regarding they safety [20,28]. Among the commonly reported adverse effects of EOs are sensitization, irritation, photosensitization, dermatitis, neurotoxicity, organ toxicity, as well as endocrine imbalances [29]. Due to their low molecular weight, lipophilicity and protein binding ability, they can easily cross also the placenta, arriving to the fetal circulation and causing fetotoxicity [30,31,32]. Therefore, their safety profile in humans should be closely monitored and more intensively investigated, as what is currently known about the adverse effects of EOs is derived from non-systematic investigation and absence of spontaneous reporting systems, contrary to authorized medicinal products [29].

The purpose of the present article is to review the main pathogens classified by the WHO as critical in terms of current AMR, summarizing the most important aspects of resistance mechanisms to classical antibiotic therapy versus the latest findings regarding the efficacy of alternative essential oil therapy. Table 3 summarizes the effect EOs have on bacterial strains categorized as critical in terms of priority by WHO.

## 2. WHO Critical Priority Pathogens

### 2.1. Carbapenem-Resistant Acinetobacter baumannii

**Figure 1:**
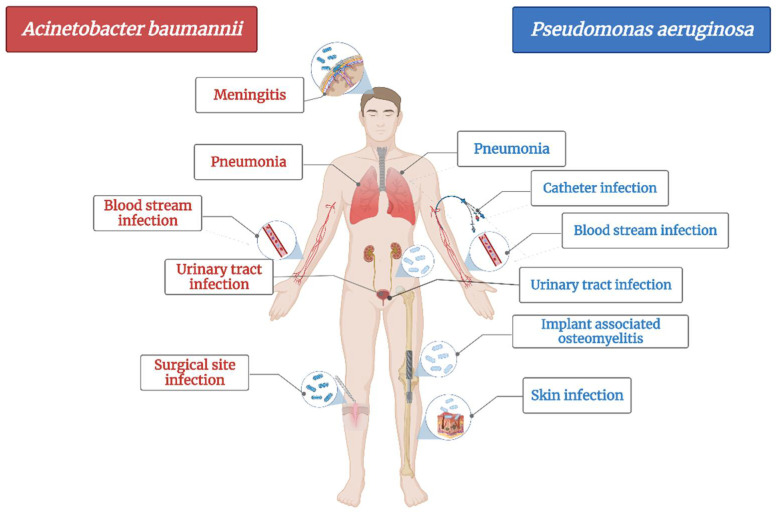
The main diseases induced by *Acinetobacter baumannii* and *Pseudomonas aeruginosa*.

*A. baumannii* is a cause of serious healthcare associated infections. Some clinical mani-festations are pneumonia, bloodstream infections, infections of lower respiratory tract, urinary tract and wounds, skin infections, meningitis, osteomyelitis and endocarditis (Figure 1) [72]. It has the ability to survive on abiotic surfaces and against disinfectant, which turns it into a “successful” nosocomial pathogen [73]. Plasticity of *Acinetobacter* spp. led to its rapid evolution in terms of developing resistance and turned it into a serious threat to hospitalized patients as treatment options are shrinking [74].

Among “ESKAPE” pathogens (*E. faecium*, *S. aureus*, *K. pneumoniae*, *A. baumanni*, *P. aeruginosa* and *E. cloacae*), *A. baumannii* has been classified as being one of the most severe bacteria in terms of antimicrobial resistance (considered nowadays to be multidrug-resistant (MDR), extensively drug-resistant (XDR), and even pandrug-resistant strain (PDR)) as it shows resistance for almost all first-line antibiotics used for healthcare associated infections (problem that has emerged since the late ’70s) [72,75].

Hospitalized patients have a higher risk of infection with *A. baumanii* due to its ability to penetrate through skin and respiratory devices [72]. The most common healthcare associated infection induced by *A. baumannii* is pneumonia, encountered mainly in patients in the intensive care unit as well as in immunocompromised ones [72,73,75].

*A. baumannii* strains can develop multiple mechanisms of antibiotic resistance. Currently, these strains are resistant to the broad-spectrum β-lactam antibiotics, carboxypenicillins, third generation of cephalosporins, and most recently to carbapenems [73]. Colistin and tigecycline have been reported to still be effective on MDR strains, alone or in combination, with some exceptions [73,76].

An important virulence factor in *A. baumannii* is the outer membrane protein (OmpA), which increases cell death by targeting mitochondria and the nucleus [72,75]. In addition, pure OMP38 induces apoptosis of human monocytes and epithelial cells [72,75]. Apoptosis of epithelial cells can reduce the surface of the mucosa and provide a way for bacteria to penetrate deeper into the tissues. Biofilms form on surfaces, and their matrix is composed of carbohydrates, nucleic acids, proteins, and other macromolecules [77]. The biofilm protects bacteria from environmental damage by host responses, antibiotics, detergents and disinfectants. Therefore, biofilms contribute to prolonged and more severe bacterial effects [77]. Biofilm-producing *A. baumanii* strains have a higher survival rate than those without biofilm. These biofilm-producing strains showed a 10–13-day survival rate on dry, untouched surfaces in intensive care units compared to other Gram-negative bacteria. In addition, these strains are able to survive on hospital bed rails and in humid environments [77].

In a study conducted in 2019, the microscopic analysis of biofilm showed that the inhibition of biofilm formation is correlated with the duration of treatment and the dose of antibiotic administered. The inhibition was much more visible at longer administration and at a higher concentration [78].

Numerous EOs have been shown to elicit antimicrobial activity and potency on *A. baumanii* strains and could be therefore seen as antibiotic alternatives and adjuvants in treating these infections [25,26,27,28,29,30]. Table 3 presents all the papers published from 2016 till present regarding the action of different EOs on *A. baumannii* strains, as well as other MDR strains.

Anchana SR et al., in 2021, evaluated the in vitro antibiofilm effect of *Ocimum sanctum* L. EO on 73 strains of carbapenem-resistant *A. baumannii*, by targeting csgA gene. The biofilm assay results highlighted a 58.9% high-grade, 31.5% low-grade and the rest (9.58%) non-biofilms formers. The minimum biofilm inhibitory concentration (MBEC50%) was 25 mcL and MBEC90% = 50 mcL, making *O. sanctum* L. EO (through its main active ingredient: benzofuran) effective in targeting this gene in carbapenem-resistant *A. baumannii* strains [33].

Two articles were found regarding the effect of *Origanum vulgare* L. EO on some of WHO List of critical pathogens [34]. Detailed information is presented in Table 3. *Origanum vulgare* L. EO was found to induce destabilization and rupture of bacterial cell membrane, and thus, apoptosis, in *A. baumannii*—metallo-β-lactamase and carbapenemase producer strains (clinical isolates) by the study performed by Amaral SC et al., in 2020. Moreover, associated with polymyxin B, it induced a synergic activity (FICI: 0.18–0.37; checkerboard assay) with a 16 fold reduction in polymyxin’s minimum inhibitory concentration (MIC) [34]. The Fractional Inhibitory Concentration Index (FICI) is a mathematical expression, used for describing the effect of antimicrobial agent combinations (e.g., additive, synergistic, antagonistic effects) [79]. It is defined as the MIC of drug A, in the presence of drug B, divided by the MIC of drug A alone (and vice versa). This concept gives an indication of the degree of drug interaction (e.g., synergy: FICI ≤ 0.5; no interaction: FICI > 0.5–4; antagonism: FICI > 4) [79].

Three studies were found regarding *Mentha* spp.’s antibacterial effect on carbapenem-resistant *A. baumannii* strains [36,39,42]. In the article published by Rinaldi F and colleagues in 2020, the effect of two EOs (*Thymus vulgaris* L. and *Syzygium aromaticum* L.) chitosan coated nanoformulations was analyzed [38]. Both intranasal formulations manifested excellent antibacterial properties against carbapenem-resistant—*A. baumannii* and *K. pneumoniae* strains, with a MIC/MBC: 0.03% *v*/*v*. A remarkable antibacterial, concentration-dependent effect was observed in *T. vulgaris* L. against both types of bacteria. When the two main phytocompounds of the EOs, thymol and eugenol, were analyzed separately, they showed an unpredicted performance, suggesting the fact that the potent antimicrobial activity of *T. vulgaris* L. is attributed to the synergic actions of all active constituents of an EO, and not just one. *S. aromaticum* L. chitosan coated nanoformulations induced a bactericidal effect against *A. baumannii*-carbapenem-resistant strains after 2 h at a conc. of 0.125% *v*/*v* and after 6 h, at a conc. of 0.06% *v*/*v*, with absence of bacterial growth. In contrast, *T. vulgaris* L. nanoformulations induced a more rapid bactericidal effect, after 2 h incubation at a conc. of 0.06% *v*/*v*. Therefore, both EOs chitosan nanoformulations make them promising solutions against MDR bacterial strains of clinical concern, as for most of the available drugs, blood–brain barrier is the major limiting factor for drugs to reach subarachnoid space [38].

### 2.2. Carbapenem-Resistant Pseudomonas aeruginosa

Likewise, *Pseudomonas aeruginosa* is also an opportunistic pathogen, the most common bacteria responsible for healthcare associated infections (Figure 1) and ventilator-associated pneumonia. It mainly affects cystic fibrosis patients and immunocompromised people and less often, healthy individuals [80]. Based on the fact that its genome is relatively large compared with other MDR strains, it has an enhanced capacity of coding regu-latory enzymes involved in the metabolism, transportation and efflux of organic substances, making it a bacterium with high adaptability and versatility to environmental changes [81]. Therefore, just as *A. baumannii*, it has developed the ability to resist most of the available antibiotics [82]. It is currently resistant to β-lactams, aminoglycosides and quinolones [80].

*P. aeruginosa* resistance to classical antibiotics has been classified and divided in three main types: intrinsic (by decreasing the outer membrane permeability, or over-expression of efflux pumps—which expel the antibiotic out of the cell or production of enzymes that inactivate the antibiotic), acquired (horizontal transfer of resistance genes = plasmids carrying genetic materials or mutations) and adaptive (formation of biofilm in the patient’s lungs that prevents antibiotic penetration and which induce prolonged and recurrent infections) [80,83]. Moreover, the outer membrane of Gram-negative bacteria is another limiting factor for antibiotic penetration, as it acts as a selective barrier, planted with porins [80,82]. More specifically, the outer membrane of *P. aeruginosa* has a 12–100× decreased permeability compared with that of *E. coli* [84].

*P. aeruginosa*’s resistance to broad-spectrum drugs such as carbapenems and cephalosporins is due to its ability to adapt by reducing the number of nonspecific porin proteins and replacing them with specific channels with low permeability to toxic chemicals [81]. Many carbapenem-resistant *P. aeruginosa* strains have been shown to be deficient in OprD porin (which is involved in antibiotic uptake and which contains the biding sites for carbapenems) [81].

Multidrug efflux pumps contribute also to its antibiotic resistance: they expel toxic and antimicrobial materials out of the bacterial cell. The four well known active multidrug efflux pumps of *P. aeruginosa* are MexAB-OprM, MexXY/OprM(OprA), MexCD-OprJ, and MexEF-OprN [80,83,85].

The long presence of *P. aeruginosa* in clinical settings is attributed to the formation of biofilms on lung epithelial cells surfaces, through the production of DNA, proteins and exopolysaccharides [80,82]. These biofilms are characterized by effective cell-to-cell communication methods, known as quorum sensing. Three main quorum sensing systems (LasR, RhlI-RhlR, PQS-MvfR) are known contributors to the formation of mature and differentiated types of *P. aeruginosa* biofilms [80,82].

Modifications in the lipopolysaccharide (the central component of the Gram-negative bacterial membrane), production of bacterial enzymes (β-lactamases, metallo-β-lactamases, aminoglycoside modifying enzymes) as well as mutations are other mechanisms by which this pathogen can acquire resistance to classical antibiotics [86].

Various EOs are effective against carbapenem-resistant *P. aeruginosa*, as shown in Table 3. Oliva A et al., investigated, in the study published in 2018, the antibacterial effect of *Melaleuca alternifolia* L., tea tree oil (TTO) (used alone or in combination with classical antibiotics) against several MDR or PDR microorganisms (including carbapenem-resistant *P. aeruginosa, A. baumannii, K. pneumoniae,* as well as methicillin-resistant *S. aureus*), in both liquid and vapor phases. TTO expressed potent antibacterial activity, with MIC/MBCs of 0.25%/0.25% *v*/*v* for carbapenem-resistant *A. baumannii, K. pneumoniae* and of 1%/1% *v*/*v* for carbapenem-resistant *P. aeruginosa*. The MIC/MBC of TTO for methicillin-resistant *Staphylococcus aureus* (MRSA) was 0.5%/2% *v*/*v*. For all tested strains, an absence of bacterial growth was observed after 24 h incubation-period. TTO expressed a potent synergistic activity at sub-inhibitory concentrations with cefazolin (lowering the MIC from 32 to 1 mcg/mL), oxacillin (from 64 to 2 mcg/mL) and with amikacin against MRSA. Regarding the synergistic activity expressed with the tested Gram-negative strains, it showed a good bactericidal action with meropenem, amikacin and colistin. Therefore, the authors postulated that TTO could be taken into account as a possible non-conventional inhalation therapy for lung infections (e.g., caused by carbapenem-resistant *A. baumannii*) or other treatment regimens (used alone or in combination with classical antibiotics) against MDR and PDR strains [44].

### 2.3. Carbapenem-Resistant and Extended Spectrum Beta-Lactamase (ESBL)-Producing Enterobacterales

#### 2.3.1. Carbapenem-Resistant and ESBL-Producing *Klebsiella pneumoniae*

**Figure 2:**
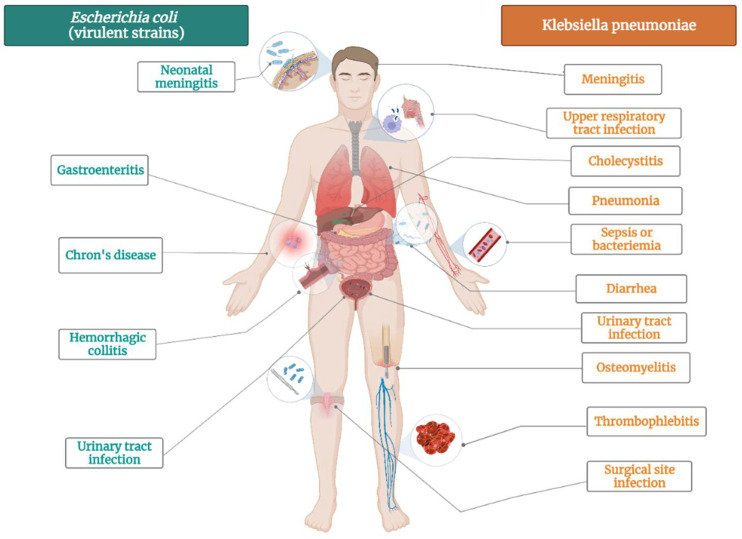
The main diseases induced by *Escherichia coli* virulent strains and *Klebsiella pneumoniae*.

*K. pneumoniae* is bacteria that commonly colonizes animal mucous membranes, from human to horses and swine. The environment represents a reservoir for human acquisition of this bacteria, given that *K. pneumoniae* is frequently found in water, sewage, soil, and plant surfaces [87]. There several strains of this pathogen: opportunistic, hypervirulent and MDR *K. pneumoniae* strains [88]. Opportunistic *K. pneumoniae* strains commonly colonize the gastrointestinal tract and might have a role to play in chronic diseases such as inflammatory bowel disease and colorectal cancer (Figure 2) [89]. In vulnerable patients, *K. pneumoniae* can also cause extraintestinal infections, such as urinary tract infection, pneumonia, bloodstream infections, wound or surgical site infections and sepsis (Figure 2) [90]. Opportunistic strains are generally acting in healthcare settings, affecting newborns, elderly, immunocompromised and hospitalized patients [90]. Other strains of *K. pneumoniae* are hypervirulent, infecting healthy people in community settings. They cause severe infections, such as pyogenic liver abscess, endophthalmitis, and meningitis [91].

Regarding the virulence of *K. pneumoniae*, so far four pathogenicity factors have been identified: pili, capsule, lipopolysaccharides (LPS), and siderophore [92,93]. These factors are chromosomally encoded and represent the basic requirements for establishing opportunistic infections. Type 1 and type 3 pili promote bacterial adhesion to epithelial, immune cells, and abiotic surfaces [91]. LPS modifications make it difficult for *K. pneumoniae* to be recognized by the host cell [94]. *K. pneumoniae* needs to acquire iron from the environment in order to thrive during infection. This is accomplished through the secretion of siderophores—molecules with a higher affinity for iron than host proteins—such as enterobactin, yersiniabactin, salmochelin, and aerobactin [95]. Elevated virulence through capsule modifications is obvious within the hypervirulent strains, associated with hypermucoviscosity. This is due to increased capsular polysaccharide production, mediated by RmpA and/or RmpA2 [91]. Moreover, *K. pneumoniae* is capable of forming biofilms—thick outer layers that favors bacterial attachment to surfaces. This asset also gives protection against ABs penetration, thus reducing their effects [96].

In terms of AMR, *K. pneumoniae* is naturally resistant to ampicillin due to the presence of the SHV-1 penicillinase in its chromosome. Resistance to other ABs occasionally occurs through chromosomal mutations, generally via horizontal gene transfer [97]. *K. pneumoniae* is increasingly developing resistance genes for aminoglycosides, quinolones, β-lactams, carbapenems, polymyxin and tigecycline. Aminoglycoside resistance is related to modification of cell permeability and the presence of aminoglycoside-modifying enzymes genes [98,99]. It has been reported that KpnO pore proteins, as well as KpnEF efflux pump systems and AcrAB-TolC, are directly involved in blocking aminoglycosides [98,99]. The primary Quinolone resistance mechanism is represented by the mutation of gyrA-gyr B subunit of DNA gyrase and parC-parE subunit of topoisomerase IV. Changes in cell permeability are also involved, including overexpression of the multidrug efflux pump gene acrAB, non-alteration of kdeA and OmpK36 deficiency [100,101,102,103,104]. The main mechanism of polymyxin resistance is target modification by chromosomal modifications. *K. pneumoniae* is able to change the structure of lipopolysaccharides in its membrane, thus affecting polymyxin binding through negative ion reduction [105]. Tigecycline resistance is acquired through modifications of the 30S and 16S ribosomal unit targets, along with changes in cell permeability, overexpression of efflux pumps AcrAB-TolC and OqxAB and changes in the expression levels of their regulators (RamA, RamR, RarA, and AcrR) [106].

*K. pneumoniae* encodes class A β-lactamase enzyme SHV in the core gene blaSH, which often suffers mutations and is routinely getting integrated into mobile genetic elements [107]. This accessory AMR gene is then horizontally transferred to other strains, resulting in ESBL activity and conferring resistance to third-generation cephalosporins and even carbapenems. Thus, *K. pneumoniae* is a crucial entry point of AMR genes into the *Enterobacterales* family [107]. In terms of human morbidity and mortality, carbapenem-resistant *K. pneumoniae* represents the fastest growing AMR threat in Europe [108]. Carbapenem-resistance is mediated by two primary mechanisms. The first one involves β-lactamases production and reduction in membrane permeability. β-lactamases, such as AmpC cephalosporinase (DHA-1 or CMY-2) or ESBL (CTX-M-2), are able to hydrolyze cephalosporins [109]. The second mechanism is mediated by the production of a β-lactamase capable of hydrolyzing most β-lactams antibiotics including carbapenems. These β-lactamases are called carbapenamases and can be divided into: *K. pneumoniae* carbapenemase (KPC), metallo-β-lactamases (VIM, IMP, NDM), and OXA-48 type enzymes [110]. Convergence of carbapenem resistance with hypervirulence results in super virulent strains, associated with high mortality rates [110]. In 2018, a first report indicated that carbapenem-resistant *K. pneumoniae* has acquired a virulence plasmid from hypervirulent *K. pneumoniae* strains [111].

#### 2.3.2. Carbapenem-Resistant and ESBL-Producing *Escherichia coli*

*E. coli* is not only the most prevalent commensal inhabitant in gastrointestinal tracts, but it is also one of the most important pathogens [112]. It can cause both intestinal pathologies (various forms of enteritis diarrhea, including hemolytic and uremic syndrome) and extraintestinal pathologies (urinary tract infections, diverse intraabdominal, pulmonary, skin and soft tissue infections, newborn meningitis and bacteremia) (Figure 2) [113]. *E. coli* is highly used in biological sciences, medicine, and industry, being the most popular microorganism in the field or recombinant DNA technology [114]. It is an important host organism in biotechnology, due to ease of handling, availability of the complete genome sequence, and its ability to grow under both aerobic and anaerobic conditions [114].

So far, eight *E. coli* pathotypes causing disease in humans have been described, six causing intestinal and two extraintestinal infections. The six intestinal pathotypes are: enteropathogenic, enterohaemorrhagic, enterotoxigenic, enteroaggregative, enteroinvasive and diffusely adherent *E. coli*. Extraintestinal pathogenic *E. coli* is divided into uropathogenic and meningitis-associated *E. coli* [115]. Among *E. coli* virulence, the biofilm formation contributes to the occurrence of infections and makes their eradication difficult. For instance, catheter-associated urinary tract infections—one of the most healthcare associated infections—are associated with the biofilm formed by *E. coli* on catheters [116]. Porins have also an important role to play in AMR, especially OmpA, OmpC and OmpF [117].

*E. coli* is very effective in acquiring resistance genes through horizontal gene transfer. Some of these genes are coding 16S rRNA methylases, inactivating aminoglycosides or plasmid-mediated quinolone resistance genes, conferring resistance to fluoroquinolones [118]. Other genes encode for ESBL, thus conferring resistance to broad-spectrum cephalosporins. This includes the ubiquitous class A ESBL enzymes, as well as class C *β*-lactamases—AmpC-type enzymes (CMY, DHA, ACC)—responsible for high-level resistance to cephalosporins [119].

Last but not least, a growing number of *E. coli* strains are able to produce carbapenamases such as class A KPC, class B metallo-β-lactamases (NDM, VIM and IMP) and class D OXA-48 [120]. These enzymes are encoded by *blaKPC, blaNDM, blaVIM, blaIMP*, and *blaOXA* genes present in both the chromosome and the plasmid [121]. Carbapenamases are associated with difficulties in detection and treatment failure, especially OXA-48-like enzyme [122]. Resistance to carbapenems is also mediated by mutations in membrane porins (especially Omp*C* and Omp*F*) and overexpression of efflux pumps [19].

The studies we found regarding the antimicrobial and antibiofilm activity of EOs on carbapenem-resistant and ESBL-producing *Enterobacterales* strains are presented in Table 3. The EOs were tested either as food additives (*Thymbra capitata* (L.) Cav.EO was proposed as food preservative for meat by Aouadhi C et al., 2022 [50]; *Artemisia herba-alba* Asso. and *Thymus algeriensis* Boiss. and Reut. EOs were proposed as natural antibacterial agents for milk shelf life as they showed a good antimicrobial activity against ESBL-producing *Enterobacterales* strains isolated from raw milk in the study performed by Sara M et al., 2021 [70]) or even substitutes for classical antibiotics in animal sector (poultry) in the study performed by Shrivastav A et al., 2019 on *Syzygium aromaticum* L. EO [55].

The studies performed by Tebrun W et al., 2020 and Motola G et al., 2020 analyzed the efficacy of several EOs as possible disinfection methods on eggshell samples [52,54]. In the study performed by Motola G et al., in 2020, from the six methods that were tested, only five of them (formaldehyde gassing; hydrogen peroxide + alcohol spray; EOs spray; peracetic acid foam and low energetic electron radiation) managed to decrease or completely eliminate the ESBL-producing *E. coli* strains. In contrast, the EOs spray that was used as cold fog could only partially reach the expected efficacy point [52].

EOs nanoformulations were also tested against different MDR strains. The study performed by Krishnamoorthy R et al., 2018 highlighted the antimicrobial activity of *Cleome viscosa* L. essential oil nanoemulsion (droplet size: 7 nm and 1:3 (*v*/*v*) oil: surfactant ratio) against ESBL-producing *E. coli* strains, *K. pneumoniae*, and *P. aeruginosa*, as well as against MRSA. The EO nanoformulation inhibited the drug efflux mechanism by damaging the cell membranes and walls of the tested strains (the active phytocompounds of the EO being: β-sitosterol, demecolcine, campesterol, and heneicosyl formate) [53].

*Origanum onites* L. EO was proposed as a possible alternative to synthetic antibacterial drugs for its antibacterial activity on ESBL-producing *E. coli* strains, after testing also its toxic effects and risks of irritation at therapeutic doses in humans, by the study performed by Kaskatepe B et al., 2017 [56], although there are also other species of the genus *Origanum* with these properties [123].

Vasquez NM et al. reported, in 2020, the antibiofilm activity of eucalyptol (the main constituent of rosemary volatile oil) on ESBL-producing *E. coli* strains isolated from urinary samples of adult patients [71]. Its bactericidal activity of eucalyptol had been already reported by the same team on *E. coli* ATCC 35218 strains in 2013 (MIC: 0.8% (*v*/*v*)) [124]. In the study, 1,8-cineole presented a concentration-dependent and a time-dependent antibiofilm activity against the biofilms that were pre-formed [71]. After only 1 h of treatment with 1% (*v*/*v*) a 3-log reduction in the viable biofilm cells was noticed, suggesting an important bactericidal activity [71].

Both studies performed by de Souza et al., 2021 and Dhara L et al., 2020 evaluated the in vitro as well as in vivo effects (animal model) of carvacrol and cinnamaldehyde and eugenol, respectively, on different carbapenem-resistant or ESBL-producing *Enterobacte-rales* strains. The in vitro antimicrobial activity of carvacrol against carbapenem-resistant *K. pneumoniae* was observed in all isolates, with a bactericidal activity observed within 4 h [62]. The in vivo antibacterial effect was showed on a mouse model of carbapenem-resistant *K. pneumoniae* infection. Carvacrol treatment increased survival and significantly decreased the bacterial load in the peritoneal lavage of the mouse. Moreover, the animals under carvacrol treatment expressed a decrease in number of leukocytes and an increase in number of platelets, but the values were within the normal range, when compared with the control group of animals [62].

The study performed by Dhara L. et al., in 2020, described the antibacterial activity of eugenol and cinnamaldehyde against ESBL producing and quinolone resistant *Enterobacterales*. Moreover, the in vivo toxicity of these EOs was investigated, in order to observe their pharmacological potential as future therapeutical solutions. Both EOs manifested good antimicrobial activity against the tested strains (data presented in Table 3), as well as good safety profiles (toxicological and behavioral effects), evaluated through a 14-day study on hematological and toxicological analyses after oral ingestion of eugenol (7.34–70 mg/kg) and cinnamaldehyde (0.91–10 mg/kg) [63].

The same team of Dhara L. and colleagues, investigated in the study published in 2020 the antimicrobial effect of cinnamaldehyde used alone or in combination with cefotaxime/ciprofloxacin. Synergism of cinnamaldehyde with cefotaxime was observed. Moreover, cinnamaldehyde managed to decrease MIC of cefotaxime and ciprofloxacin up to 1024×, with a bactericidal and synergistic effect observed after 24 h [64].

Qian W. and colleagues showed, in the study published in 2019, the antimicrobial effect of eugenol, as well as its effect on biofilm formation and biofilm-associated gene expression of carbapenem-resistant *K. pneumoniae* strains. Eugenol managed to deteriorate cell membrane by decreasing ATP concentration, reducing its pH, increasing hyperpolarization and by stimulating its permeability [68].

## 3. Materials and Methods

The first step in the elaboration of the present review was the selection of the topic. Based on the current issues of AMR and the WHO list of bacteria that require imperative new solutions of treatment, three researchers (V.B., C.D and D.M.) conducted a search in the scientific literature in order to gather more data regarding this topic. They noticed that updated reviews on the efficacy of EOs on different MDR strains were lacking; therefore, they decided to conduct a review on this subject. The aim was to identify the latest findings regarding the efficacy of EOs on the WHO list of critical pathogens (also known as Priority 1 pathogens). A working team was established with members having experience in the area of interest: clinical laboratory, microbiology, botany, phytotherapy and clinical pharmacology.

**Figure 3:**
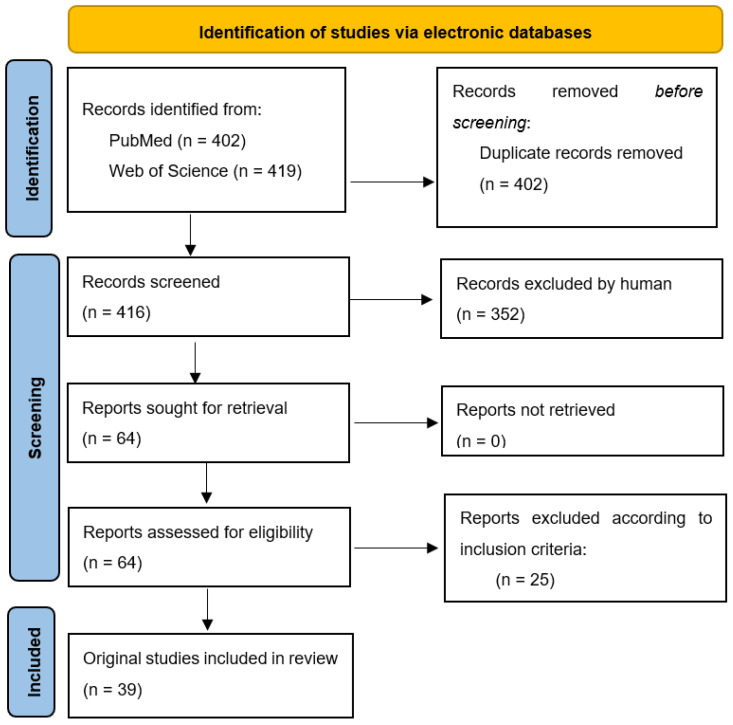
Prisma flow chart for systematic reviews [125].

From March 2022 to end of April 2022, authors conducted independent research on 2 electronic databases (PubMED and Web of Science) in order to identify original articles describing the activity of EOs on each critical pathogen mentioned in the WHO’s list (Figure 3). The keywords used to perform this research were the following: *Name of WHO critical pathogen* AND *antibiotic resistance* AND *essential oils* (e.g., *A. baumannii* AND carbapenem-resistant AND essential oils). Articles that were published starting from January 2016 were included (in vitro as well as in vivo studies). Inclusion criteria: WHO Priority 1 pathogens; the specific type of resistance mentioned by WHO; articles discussing the antibacterial and/or antibiofilm efficacy of a minimum EO; from 2016 to 2022. A template (same table head as in Table 3) was built by the working team for data extraction.

After each independent researcher extracted the required data, a face-to-face meeting was conducted in order to discuss the highlights of each article. Data validation and disagreements were solved by a researcher with expertise in microbiology (D.M.).

The mechanism of developing resistance for each pathogen was documented by a B.B. and a graphic representation of the main clinical diseases induced by each bacterium was made by A.L., using BioRender.com (accessed on 1 June 2022).

## 4. Conclusions

As the golden era of classical ATB seems to be over due to the occurrence of MDR strains, medical practitioners must be opened to new approaches in treating infections. In this regard, healthcare associations need to come up with innovative measures to prevent and slowdown of AMR and establish a global antimicrobial guide that completely tackles this global health issue. At the same time, the entire scientific community needs to conti-nuously work on investigating alternative solutions to fight AMR. There are some directions that are currently under exploration, such as identifying new compounds with different mechanisms of actions; developing alternative administration routes for the existing compounds; and working on the formulations through adjusting the adjuvant substances. However, one key tool in fighting AB resistance might actually lie in the thorough understanding of the underlying mechanism of AMR. Knowledge about how and when resistance occurs can help minimize the emergence of MDR pathogens, since new ABs are developed at a slow pace. Thus, the real challenge nowadays is how to make best use of the available assets, including technology, knowledge, and actives such as classical ABs and EOs. Little is known regarding the efficacy and safety of EOs in humans, given that most of the existent research being conducted in vitro. Consequently, much more research is needed in order to have a clear picture on the benefits, as well as the risks of using EOs as alternative to the current, classical antimicrobial therapy, in humans. Moreover, finding efficacious and safe synergistic drug combinations could be another solution for the current challenges of AMR.

## Figures and Tables

**Table 1:** European prevalence of critical priority bacteria that acquired 3rd generation cephalosporin-resistance [6].

Bacteria	% of Bacteria Resistant to 3rd Generation Cephalosporins	Country
*Klebsiella pneumoniae*	79.1	Bulgaria
	74.5	Greece
	67.9	Romania
	63.0	Poland
	54.7	Cyprus
	54.4	Slovakia
*Escherichia coli*	41.4	Bulgaria
	29.8	Cyprus
	27.1	Slovakia
	26.4	Italy
	24.1	Latvia
	21.9	Greece

**Table 2:** European prevalence of critical priority bacteria that acquired carbapenem-resistance [6].

Bacteria	% of Bacteria Resistant to Carbapenems	Country
*Acinetobacter* spp.	96.4	Croatia
	94.6	Greece
	93.3	Romania
	91.1	Lithuania
	82.9	Bulgaria
	82.7	Latvia
*Pseudomonas aeruginosa*	48.9	Slovakia
	43.9	Romania
	42.9	Bulgaria
	35.7	Greece
	33.8	Hungary
	30.3	Croatia
*Klebsiella pneumoniae*	66.3	Greece
	48.3	Romania
	29.5	Italy
	28.1	Bulgaria
	19.8	Cyprus
	19.1	Croatia
*Escherichia coli*	0.8	Bulgaria
	0.7	Romania
	0.5	Greece
	0.5	Italy
	0.4	Spain
	0.2	Portugal

**Table 3:** The effects of different EOs on WHO critical priority pathogens.

Study Team and Year	Bacterial Strain	Essential Oil(s)/ Pure Phytocompound	Method(s)	Results	Reference
**Carbapenem-Resistant *Acinetobacter baumannii***					
Anchana SR et al., 2021	carbapenem-resistant *A. baumanni*	*Ocimum sanctum* L. compunds	Semi-quantitative adherent bioassay PCR amplification csgA gene sequencing Molinspiration assessment Cristal violet assay	Benzofuran from *Ocimum sanctum* L. EOs can be effectivein targeting csgA gene of carbapenem-resistant *A. baumanni* strains. Minum biofilm inhibition conc (MIC) of 50% was observed at 25 mcL and at 90%, 50 mcL.	[33]
Amaral SC et al., 2020	carbapenem-resistant *A. baumanni*	*Origanum vulgare* L. EO	PCR for gyrB and blaOXA-51-like genes Flow cytometry Checkerboard assay	Carvacrol (71%) showed antibacterial effect against all Ab-MDR tested strains (MIC: 1.75–3.50 mg mL^−1^) Synergistic interaction with polymyxin B (16× ↓ in polymyxin B MIC)	[34]
Vasconcelos NG et al., 2019	*A. baumanii*; *K. pneumoniae;* carbapenem-resistant *Serratia marcescens*	*Origanum vulgare* L. EO	Disk-diffusion Microdilution Time kill assays	MIC: 0.015% *v*/*v* for *A. baumannii*, MIC: 0.059% *v*/*v* for *K. pneumoniae* and *S. marcescens* Decrease in cell count: after 4 h treatment	[35]
Bekka-Hadji F et al., 2022	imipenem-resistant *A. baumannii*; MRSA	*Mentha pulegium* L. *Artemisia herba alba* Asso.	Disk diffusion method Microdilution broth assay	*Mentha pulegium* L. EO was found to containe pulegone (74.8%) and neoisomenthol (10.0%). *Artemisia herba alba* Asso. EO was found to contain maily camphor (32.0%), α-thujone (13.7%), 1,8-cineole (9.8%), β-thujone (5.0%). Synergistic, antagonic or neutral effects of EOs with antibiotics were observed. The strongest: *M. pulegium* L. + amikacin for Imipenem-resitant *A. baumannii*. *P. aeruginosa* strains were found to be resistant to these EOs.	[36]
Oliva A et al., 2020	carbapenem-resistant *A. baumannii*, *K. pneumoniae* and *P. aeruginosa*; MRSA; *E. coli* (ATCC 25922); *C. albicans* (ATCC 14053)	*Helichrysum italicum* (Roth) G. Don fil.	Dilution and disk diffusion method	The EO was analyzed in both liquid and vapour phases. Bactericidal/Fungicidal effect of EO characterized maily by β-eudesmene (21.65%) and β-bisabolene (19.90%) was observed at a concentration of 5% *v*/*v* against carbapenem- resistant *A. baumannii* and *C. albicans.*	[37]
Rinaldi F et al., 2020	carbapenem-resistant *A. baumannii*, *K. pneumoniae* and *P. aeruginosa*; MRSA; *E. coli* (ATCC 25922)	*Thymus vulgaris* L. *Syzygium aromaticum* L. EOs chitosan nanoemultions	Macro dilution broth methods Disk diffusion assay Time kill assay	Both EOs nanoformulations presented a MIC/MBC for *A. baumannii*- and *K. pneumoniae*—carbapenem resistant of 0.03% *v*/*v*. Amazing concentration-dependent, antibacterial effect was observed for *T. vulgaris* L. nanoformulations against the two strains mentioned above. Effective intranasal formulations (promising therapeutical solutions).	[38]
Mahmoudi H et al., 2020	carbapenem-resistant and fluoroquinolones-resistant *A. baumannii*	*Mentha longifolia* (L.) Hudson (Menthol)	PCR method Microbroth dilution method	MIC decreased 4× when *Mentha longifolia* (L.) Hudson EO was associated with ciprofloxacin and 8× when associated with imipenem. Menthol EO + imipenem reduced the resistance to imipenem up to 16× in 90% of the analyzed strains.	[39]
Kaskatepe B et al., 2016	carbapenem-resistant *A. baumannii* and *P. aeruginosa*	Commercial cinnamon oil- the exact species not specified	Disk diffusion method	*P. aeruginosa* MIC: 0.0019 mL/mL, 21 mm zone diameter	[40]
Laktib A et al., 2021	carbapenem-resistant *A. baumannii*	*Lavandula mairei* Humbert	Disk diffusion Broth microdilution method	All the tested strains showed sensitivity to the EO. MIC: 0.39–3.125 mcL/mL IDZ: 28.67–40 mm	[41]
Muntean D et al., 2019	carbapenem-resistant *A. baumannii*, *P. aeruginosa* and *Enterobacterales*	*Mentha × piperita* L.	Agar disk diffusion method Microdilution method	MIC: <20 mg/mL for *S. aureus*, *E. coli*, *P. mirabilis* MIC: >40 mg/mL for *K. pneumoniae*, *P. aeruginosa* and *A. baumannii*	[42]
**Carbapenem-Resistant *Pseudomonas aeruginosa***					
Patterson JE et al., 2019	MRSA; carbapenem-resistant and ESBL-producing *Enterobacterales*; *MDR* (including carbapenem- resistant) *P. aeruginosa*	*Cinnamomun zeylanicum* L. *Szygium aromaticum* L. *Cymbopogon flexuosus* L. *Origanum vulgare* L. *Rosmarinus officinalis* L. *Thymus vulgaris* L. *Melaleuca alternifolia* (Maiden and Betche) *Leptospermum scoparium* J. R. et G. Forst 1 EO blend (cinnamon, clove, lemon, eucalyptus, rosemary)	Disk diffusion method	Cinnamon bark EO had the largest zone of inhibition against *P. aeruginosa* (18 mm). The largest inhibition’s zones for *Enterobacterales* spp. were observed for cinnamon bark, oregano and thyme.	[43]
Oliva A et al., 2018	carbapenem-resistant *P. aeruginosa*, *A. baumannii and* *K. pneumoniae*; *ESBL-producing and* *carbapenem-sensitive* *K. pneumoniae*; MRSA	*Melaleuca alternifolia* (Maiden and Betche)	Macro dilution broth method Checkerboard assay Disk diffusion Vapour assay Time kill assay	Tea tree oil was reported to be an effective antimicrobial agent, used alone or in association with classical antibiotic therapy (ex: oxacillin against MRSA). Tea tree oil administered by inhalation could be an option for carbapenem— resistant *A. baumannii* induced pneumonia.	[44]
**Carbapenem-Resistant *Enterobacterales***					
Qian W et al., 2020	carbapenem-resistant *Enterobacter cloacae*	Citral (isomeric mixture of geranial and neral)	Agar dilution method Confocal laser scanning microscopy	MIC: 1000 mcg/mL. Potent antibacterial and antibiofilm activity was observed. Potential use as natural food preservative.	[45]
Ginting EV et al., 2021	ESBL-producing *E. coli* and *K. pneumoniae*	*Syzygium aromaticum* L. *Cinnamomum verum* J.Presl	Disk diffusion method Broth microdilution method Scanning electron microscopy	High antibacterial activity of both EOs. Clove EO MIC: 0.078% (*v*/*v*) for all bacteria. Cinnamon EO MIC: 0.039% (*v*/*v*)–0.156 (*v*/*v*) for all bacteria	[46]
Sharifi-Rad J et al., 2016	ESBL-producing *E. coli*	*Achillea wilhelmsii* C. Koch, *Echinophora platyloba* DC., *Lallemantia royleana*, *Nepeta persica* Boiss., *Pulicaria* *vulgaris* Gaertn., *Salvia* *nemorosa* L., *Satureja* *intermedia* C.A.Mey	Microdilution method Phenotypic disc confirmatory test Polymerase chain reaction for TEM gene	All tested EOs presented high antimicrobial activity	[47]
Kwiatkowski P et al., 2018	ESBL and New Delhi metallo-β-lactamase-1 (NDM-1) producing *K. pneumoniae*	*Carum carvi* L., *Foeniculum vulgare* Mill., *Mentha* × *piperita* L., *Pelargonium* *graveolens* L’Hér. *Ocimum basilicum* L., *Syzygium aromaticum* L. Merrill and Perry, *Thymus vulgaris* L., *Salvia sclarea* L., *Lavandula angustifolia* Mill.	Broth microdilution method Checkerboard method	Peppermint oil + gentamicin induced synergistic effects against all tested strains. Caraway EO + gentamicin induced synergistic effects against ESBL-strains and gentamicin-resistant strains. Association of gentamicin + thyme, basil, fennel and clary sage induced an additive antimicrobial effect.	[48]
Benameur Q et al., 2019	blaESBL-producing *Enterobacterales* strains	*Thymus vulgaris* L. alone or associated with cefotaxime	Disc diffusion assay PCR Checkerboard test	*Thymus vulgaris* L. EO showed increased antimicrobial activity against all tested strains (MIC: 24–40 mm/10 mcL for MDR strains and 2.87–11.5 mcg/mL for blaESBL producing strains). Synergistic activity was observed when *Thymus vulgaris* L. EO was associated with cefotaxime for blaESBL-producing *E. coli* strains, as well as an additive activity for ESBL-producing *Enterobacter cloacae*	[49]
Aouadhi C et al., 2022	ESBL-producing *E. coli*	*Eucalyptus globulus* L., *Eucalyptus camaldulensis* Dehnh., *Artemisia absinthium* L., *Myrtuscommunis* L., *Mentha pulegium* L., *Trachyspermum ammi* (L.) Sprague, *Cymbopogon citratus* (DC.) Stapf, *Thymbra capitata* (L.) Cav.	Disc-diffusion assay Broth dilution method Time-kill study Bacteriolysis	*Thymbra capitata* (L.) Cav. EOs induced the most potent inhibitory effect against *E. coli* strains (MIC: 0.02–0.78%) by reducing its viability, NaCl tolerance, enhancing the loss of 260 nm-absorbing material and sensitive to autolysis. *Thymbra capitata* (L.) Cav. EO was proposed as food preservative (especially in meat).	[50]
Saliu EM et al., 2020	ESBL-producing *E. coli;* *Salmonella typhimurium* inoculated in cecal contents of 2 weeks old broilers	Carvacrol Cinnamaldehyde Eugenol *Lactobacillus agilis* LA73 *Lactobacillus salivarius* LS1 used as feed additives (alone or in combination)	Inoculation Aerobic co-incubation	The experimental diet highlighted a lower ESBL-prevalence in the study group of animals (broilers) when EOs and *Lactobacillus* spp. were added. Horizontal gene transfer effect was less obvious.	[51]
Motola G et al., 2020	ESBL producing *E. coli*	Essential oils used as spray or cold mist disinfectant method	-	The essential oil cold fog disinfecting method managed to partly reach the expected efficacy threshold in reducing ESBL-producing *E. coli* contamination on the hatching egg.	[52]
Krishnamoorthy R et al., 2018	ESBL-producing *E. coli*, *K. pneumoniae* and *P. aeruginosa*; MRSA	*Cleome viscosa* L. essential oil nanoemulsion	Agar well diffusion method Broth dilution method	The nanoemultion was effective against the tested strains by inhibiting the drug efflux mechanism. Nanofromulation from *Cleome viscosa* L. proved to induce a broad-spectrum antimicrobial activity against the tested strains. Gram-negative ESBL-producing strains: MIC: 30 mcg/mL; MBC: 40 mcg/mL; MRSA MIC: 25 mcg/mL; MBC: 40 mcg/mL, including ATCC control strain, the nanoemulsion being more effective against Gram-positive bacteria pathogens.	[53]
Tebrün W et al., 2020	ESBL-producing *E. coli*	Essential oil spray (a mixture of *Origanum* *vulgare* L. oil and alcohol)	-	Decreased hatchability was observed for essential oil spray application on egg broiler chicks, leading it to be inappropriate in daily practice, compared to the application of hydrogen peroxide as disinfectant protocol.	[54]
Shrivastav A et al., 2019	ESBL-producing *E. coli*	*Syzygium aromaticum* L. EO and *Ocimum sanctum* L. fresh jouice	Colorimetric assay	Higher inhibitory activity observed for *S. aromaticum* L. EO on beta-lactamase enzyme of cecal samples of healthy broilers.	[55]
Kaskatepe B et al., 2017	ESBL-producing *E. coli*	*Origanum onites* L.	Disc diffusion Agar dilution method Micro-dilution method	*Origanum onites* L. EO presented antimicrobial activity against all tested strains and also inhibited microbial growth on ESBL-producing *E. coli* strains. MIC: 1.56–25 mcL/mL; 3.12–25 mcL/mL	[56]
Tadić V et al., 2017	carbapenem-resistant *K. pneumoniae*; *E. coli* (ATCC 25922); MRSA	*Sideritis romana* L. subsp. purpurea (Tal. ex Benth.) Heywood	Mueller Hinton broth	Good antimicrobial effect against MRSA with MIC: 0.076 mg/mL and MBC: 0.153 mg/mL. No effect on carbapenem resistant *K. pneumoniae* or *E. coli.*	[57]
Iseppi R et al., 2020	ESBL-producing *E. coli* and *K. pneumoniae*; Carbapenamase-producing *K. pneumoniae*; metallo-beta-lactamase-producing *P. aeruginosa*	*Melaleuca alternifolia* (Maiden and Betche), *Eucalyptus globulus* L., *Mentha* × *piperita* L., *Thymus vulgaris* L.	Agar disk diffusion assay	*Melaleuca alternifolia* (Maiden and Betche) and *Thymus vulgaris* L. EOs were the most effective on the tested strains, the first EO being effective even at low concentrations. *Melaleuca alternifolia* (Maiden and Betche) MIC: 0.5–16 mcg/mL. *Thymus vulgaris* L. MIC: 1–16 mcg/mL. Both EOs presented also a good anti-biofilm activity, making them good candidates for infections caused by these pathogens even in association with classical antibiotics.	[58]
Gāliņa D et al., 2022	ESBL-producing *E. coli*	*Thymus serpyllum* L. *Thymus vulgaris* L. *Satureja montana* L. alone or in combination with caprylic acid and sodium chloride	Broth microdilution method	The antimicrobial activity of tested EOs was superior when sodium chloride or caprylic acid was added. All tested EOs showed good antimicrobial activity against ESBL and non-ESBL-producing, as well as MDR and non-MDR *E. coli* strains.	[59]
Benameur Q et al., 2021	ESBL-producing *Enterobacterales*	*Origanum vulgare* L.	Disc diffusion assay Twofold serial dilution method	High antibacterial effect against all studied strains MIC: 0.31–5 mcL/mL, ESBL-producing strains being more responsive to EO.	[60]
Contreras-Moreno BL et al., 2016	ESBL-producing *E. coli* and *Enterobacter cloacae*; MRSA	*Pimenta racemosa* var. racemose (Mill.) of different densities	Disc diffusion agar method	Antibacterial effect on all the tested strains of nosocomial provenience. MIC: 20–400 mcL/mL.	[61]
de Souza et al., 2021	carbapenem-resistant *K. pneumoniae*	Carvacrol	Broth microdilution method Time-kill assay Mouse model of infection	Antibacterial effect on all the tested strains with eradication of all bacterial cells within 4 h. MICs/MBCs: 130–260 mg/L. The in vivo effect of carvacrol determined through a mouse model of infection induced an increased survival and a decreased bacterial load in the peritoneal lavage. Moreover, leucopenia was observed, as well as increased number of platelets when compared with the control group.	[62]
Dhara L et al., 2020	ESBL-producing and quinolone-resistant *Enterobacterales*	Cinnamaldehyde Eugenol	Broth microdilution method Murine model toxicity level	*E. coli* MIC: 7.28 mcg/mL (cinnamaldehyde); 7.34 mcg/mL (eugenol). *K. pneumoniae* MIC: 0.91 mcg/mL (cinnamaldehyde); 3.67 mcg/mL (eugenol). Good safety profiles manifested by both EOs in a murine model (Swiss albino mice).	[63]
Dhara L, Tripathi A et al., 2020	ESBL-producing and quinolone-resistant *Enterobacterales*	Cinnamaldehyde alone or in combination with cefotaxime/ciprofloxacin	Broth microdilution method Checkerboard assay Isobologram analysis Time-kill assay	Synergism of cinnamaldehyde with cefotaxime was observed. Cinnamaldehyde managed to decrease MIC of cefotaxime and ciprofloxacin up to 1024×, the bactericidal and synergistic effect being observed after 24 h.	[64]
Gore MR et al., 2021	ESBL-producing *Enterobacterales*	*Pimenta dioica* (L.) Merr. extracts	Agar dilution method	Chloroform extract induced the maximal antibacterial activity, with MBC: 2–5 mg/mL. Ampicillin MBC decreased from 10 mg/mL to 300–500 mcg/mL in the presence of *Pimenta dioica* (L.) Merr. Chloroform extracts and thus it induced a synergistic action. Eugenol was observed to be the major compound of the extract. EO induced a deformation in the bacterial cell membrane (stress and cellular damage).	[65]
Khan I et al., 2017	ESBL-producing *E. coli*	Carvacrol	Broth microdilution method	MIC: 450 mcg/mL, time-dependent effect (after 2 h it managed to completely diminish the growth in *E. coli* strains). The antibacterial effect of carvacrol on the tested strains was induced by high level of reactive oxygen species and bacterial cell membrane depolarization, leading to its disruption and release of cellular material.	[66]
Kose EO et al., 2021	carbapenem-resistant *K. pneumoniae*	Carvacrol + meropenem	Broth microdilution method Checkerboard assay Time-kill assay	Carvacrol + meropenem MIC: 32–128 mcg/mL, with a synergy between the 2 substances observed in 8 of the 25 tested strains. Cell membrane damage was observed.	[67]
Qian W et al., 2020	carbapenem-resistant *K. pneumoniae*	Eugenol	Agar dilution method	Eugenol MICs: 0.2 mg/mL. Strong inhibitory effects on biofilm formation and biofilm- associated gene expression.	[68]
Ramachandran G et al., 2020	carbapenem-resistant *K. pneumoniae*	*Camellia japonica* L.	Disc diffusion Agar well method Micro broth dilution method	MIC: 50 mcg/mL, concentration dependent effect on membrane destruction.	[69]
Sara M et al., 2021	ESBL-producing *Enterobacterales*	*Artemesia herba-alba* Asso. *Thymus algeriensis* Boiss and Reut.	Disc diffusion Microdilution methods	*K. pneumoiae* SB6 had the highest zones of inhibition. Both EOs MIC: 1.56–12.5 mg/mL. *A. herba-alba* MBC: 6.25–25 mg/m. *T. algeriensis* MBC: 12.5–25 mg/mL. Possible new natural preservative solution for dairy products, although in vivo studies are needed in order to determine their safety profile as well as their acceptability of the aroma and flavor of EOs by the consumer.	[70]
Vasquez NM et al., 2020	ESBL-producing *E. coli*	*Rosmarinus officinalis* L.	Broth microdilution method	1,8-cineole (eucalyptol) presents antimicrobial [MIC: 0.8 (*v*/*v*)] and antibiofilm activities against uropathogenic *E. coli* ESBL-producing strains. The antibiofilm activity has been reported to be concentration-dependent as well as time-dependent over pre-formed biofilm, suggesting it to be a good candidate for *E. coli* biofilm infections.	[71]

## Data Availability

All the data is presented in the present manuscript.

## Abbreviations

ABs	Antibiotics
AMR	Antimicrobial resistance
CNS	Central nervous system
EOs	Essential oils
ESBL	Extended spectrum beta-lactamase.
FICI	Fractional inhibitory concentration index
KPC	*K. Pneumoniae* carbapenemase
LPS	Lipopolysaccharides
MBEC_50%_	Minimum biofilm inhibitory concentration
MDR	Multidrug resistant
MIC	Minimum inhibitory concentration
MRSA	Methicillin-resistant *Staphylococcus aureus*
OmpA	Outer membrane protein
PDR	Pandrug-resistant
TTO	Tea tree oil
WHO	World Health Organization
XDR	Extensively drug-resistant

## References

[1] Aslam B., Wang W., Arshad M.I., Khurshid M., Muzammil S., Rasool M.H., Nisar M.A., Alvi R.F., Aslam M.A., Qamar M.U. (2018). Antibiotic Resistance: A Rundown of a Global Crisis. Infect. Drug Resist..

[2] Dadgostar P. (2019). Antimicrobial Resistance: Implications and Costs. Infect. Drug Resist..

[3] Mancuso G., Midiri A., Gerace E., Biondo C. (2021). Bacterial Antibiotic Resistance: The Most Critical Pathogens. Pathogens.

[4] Murray C.J., Ikuta K.S., Sharara F., Swetschinski L., Robles Aguilar G., Gray A., Han C., Bisignano C., Rao P., Wool E. (2022). Global Burden of Bacterial Antimicrobial Resistance in 2019: A Systematic Analysis. Lancet.

[5] World Health Organization (2017). Global Priority List of Antibiotic-Resistant Bacteria to Guide Research, Discovery, and Development of New Antibiotics. Cad. Pesqui..

[6] Surveillance Atlas of Infectious Diseases. https://www.ecdc.europa.eu/en/surveillance-atlas-infectious-diseases.

[7] Mayor S. (2005). Antibiotic resistance is highest in south and east Europe. BMJ.

[8] European Centre for Disease Prevention and Control (ECDC) (2012). Summary of the Latest Data on Antibiotic Resistance in the European Union.

[9] Elmahi O.K.O., Uakkas S., Olalekan B.Y., Damilola I.A., Adedeji O.J., Hasan M.M., dos Santos Costa A.C., Ahmad S., Essar M.Y., Thomson D.J. (2022). Antimicrobial Resistance and One Health in the Post COVID-19 Era: What Should Health Students Learn?. Antimicrob. Resist. Infect. Control.

[10] Chokshi A., Sifri Z., Cennimo D., Horng H. (2019). Global Contributors to Antibiotic Resistance. J. Glob. Infect. Dis..

[11] Martin M.J., Thottathil S.E., Newman T.B. (2015). Antibiotics Overuse in Animal Agriculture: A Call to Action for Health Care Providers. Am. J. Public Health.

[12] McEwen S.A., Collignon P.J. (2018). Antimicrobial Resistance: A One Health Colloquium. Microbiol. Spectr..

[13] Zalewska M., Błażejewska A., Czapko A., Popowska M. (2021). Antibiotics and Antibiotic Resistance Genes in Animal Manure–Consequences of Its Application in Agriculture. Front. Microbiol..

[14] Ma Z., Lee S., Jeong K.C. (2019). Mitigating Antibiotic Resistance at the Livestock-Environment Interface:A Review. J. Microbiol. Biotechnol..

[15] Aslam B., Khurshid M., Arshad M.I., Muzammil S., Rasool M., Yasmeen N., Shah T., Chaudhry T.H., Rasool M.H., Shahid A. (2021). Antibiotic Resistance: One Health One World Outlook. Front. Cell. Infect. Microbiol..

[16] World Health Organization (2015). Global Action Plan on Antimicrobial Resistance. https://apps.who.int/iris/handle/10665/193736.

[17] Butler M.S., Gigante V., Sati H., Paulin S., Al-Sulaiman L., Rex J.H., Fernandes P., Arias C.A., Paul M., Thwaites G.E. (2022). Analysis of the Clinical Pipeline of Treatments for Drug-Resistant Bacterial Infections: Despite Progress, More Action Is Needed. Antimicrob. Agents Chemother..

[18] Terreni M., Taccani M., Pregnolato M. (2021). New Antibiotics for Multidrug-Resistant Bacterial Strains: Latest Research Developments and Future Perspectives. Molecules.

[19] Murugan M.S., Sinha D.K., Vinodh Kumar O.R., Yadav A.K., Pruthvishree B.S., Vadhana P., Nirupama K.R., Bhardwaj M., Singh B.R. (2019). Epidemiology of Carbapenem-Resistant *Escherichia coli* and First Report of BlaVIM Carbapenemases Gene in Calves from India. Epidemiol. Infect..

[20] Ramsey J.T., Shropshire B.C., Nagy T.R., Chambers K.D., Li Y., Korach K.S. (2020). Essential Oils and Health. Yale J. Biol. Med..

[21] Plant R.M., Dinh L., Argo S., Shah M. (2019). The Essentials of Essential Oils. Adv. Pediatr..

[22] Wińska K., Mączka W., Łyczko J., Grabarczyk M., Czubaszek A., Szumny A. (2019). Essential Oils as Antimicrobial Agents—Myth or Real Alternative?. Molecules.

[23] Yu Z., Tang J., Khare T., Kumar V. (2020). The Alarming Antimicrobial Resistance in ESKAPEE Pathogens: Can Essential Oils Come to the Rescue?. Fitoterapia.

[24] Valerio F., Mezzapesa G.N., Ghannouchi A., Mondelli D., Logrieco A.F., Perrino E.V. (2021). Characterization and Antimicrobial Properties of Essential Oils from Four Wild Taxa of *Lamiaceae* Family Growing in Apulia. Agronomy.

[25] Khoury M., Stien D., Eparvier V., Ouaini N., El Beyrouthy M. (2016). Report on the Medicinal Use of Eleven Lamiaceae Species in Lebanon and Rationalization of Their Antimicrobial Potential by Examination of the Chemical Composition and Antimicrobial Activity of Their Essential Oils. Evid. Based Complement. Alternat. Med..

[26] Perrino E.V., Valerio F., Jallali S., Trani A., Mezzapesa G.N. (2021). Ecological and Biological Properties of *Satureja cuneifolia* Ten. and *Thymus spinulosus* Ten.: Two Wild Officinal Species of Conservation Concern in Apulia (Italy). A Preliminary Survey. Plants.

[27] Zhang N., Yao L. (2019). Anxiolytic Effect of Essential Oils and Their Constituents: A Review. J. Agric. Food Chem..

[28] Posadzki P., Alotaibi A., Ernst E. (2012). Adverse Effects of Aromatherapy: A Systematic Review of Case Reports and Case Series. Int. J. Risk Saf. Med..

[29] Vostinaru O., Heghes S.C., Filip L. (2020). Safety Profile of Essential Oils. Essential Oils-Bioactive Compounds, New Perspectives and Applications.

[30] Wojtunik-Kulesza K.A. (2022). Toxicity of Selected Monoterpenes and Essential Oils Rich in These Compounds. Molecules.

[31] Lanzerstorfer P., Sandner G., Pitsch J., Mascher B., Aumiller T., Weghuber J. (2021). Acute, Reproductive, and Developmental Toxicity of Essential Oils Assessed with Alternative In Vitro and In Vivo Systems. Arch. Toxicol..

[32] Dosoky N.S., Setzer W.N. (2021). Maternal Reproductive Toxicity of Some Essential Oils and Their Constituents. Int. J. Mol. Sci..

[33] Saishree Anchana R., Smiline Girija A.S., Gunasekaran S., Vijayashree Priyadharsini J. (2021). Detection of *CsgA* Gene in Carbapenem-Resistant *Acinetobacter baumannii* Strains and Targeting with *Ocimum sanctum* Biocompounds. Iran. J. Basic Med. Sci..

[34] Amaral S.C., Pruski B.B., de Freitas S.B., Allend S.O., Ferreira M.R.A., Moreira C., Pereira D.I.B., Junior A.S.V., Hartwig D.D. (2020). Origanum Vulgare Essential Oil: Antibacterial Activities and Synergistic Effect with Polymyxin B against Multidrug-Resistant *Acinetobacter baumannii*. Mol. Biol. Rep..

[35] Vasconcelos N.G., Croda J., Silva K.E., Motta M.L.L., Maciel W.G., Limiere L.C., Simionatto S. (2019). Origanum Vulgare L. Essential Oil Inhibits the Growth of Carbapenem-Resistant Gram-Negative Bacteria. Rev. Soc. Bras. Med. Trop..

[36] Bekka-Hadji F., Bombarda I., Djoudi F., Bakour S., Touati A. (2022). Chemical Composition and Synergistic Potential of *Mentha pulegium* L. and *Artemisia herba alba* Asso. Essential Oils and Antibiotic against Multi-Drug Resistant Bacteria. Molecules.

[37] Oliva A., Garzoli S., Sabatino M., Tadić V., Costantini S., Ragno R., Božović M. (2020). Chemical Composition and Antimicrobial Activity of Essential Oil of *Helichrysum italicum* (Roth) G. Don Fil. (Asteraceae) from Montenegro. Nat. Prod. Res..

[38] Rinaldi F., Oliva A., Sabatino M., Imbriano A., Hanieh P.N., Garzoli S., Mastroianni C.M., De Angelis M., Miele M.C., Arnaut M. (2020). Antimicrobial Essential Oil Formulation: Chitosan Coated Nanoemulsions for Nose to Brain Delivery. Pharmaceutics.

[39] Mahmoudi H., Shokoohizadeh L., Zare Fahim N., Mohamadi Bardebari A., Moradkhani S., Alikhani M.Y. (2020). Detection of AdeABC Efllux Pump Encoding Genes and Antimicrobial Effect of Mentha Longifolia and Menthol on MICs of Imipenem and Ciprofloxacin in Clinical Isolates of *Acinetobacter baumannii*. BMC Complement. Med. Ther..

[40] Kaskatepe B., Kiymaci M.E., Suzuk S., Erdem S.A., Cesur S., Yildiz S. (2016). Antibacterial Effects of Cinnamon Oil against Carbapenem Resistant Nosocomial *Acinetobacter baumannii* and *Pseudomonas aeruginosa* Isolates. Ind. Crops Prod..

[41] Laktib A., Nayme K., El Hamdaoui A., Timinouni M., Hassi M., Aitalla A., Msanda F., Bourouache M., El Yaagoubi M., Mimoun R. (2022). Antibacterial Activity of Lavandula Mairei Humbert Essential Oil against Carbapenem-Resistant *Acinetobacter baumannii*. Mediterr. J. Infect. Microbes Antimicrob..

[42] Muntean D., Licker M., Alexa E., Popescu I., Jianu C., Buda V., Dehelean C.A., Ghiulai R., Horhat F., Horhat D. (2019). Evaluation of Essential Oil Obtained from *Mentha* × *piperita* L. against Multidrug-Resistant Strains. Infect. Drug Resist..

[43] Patterson J.E., McElmeel L., Wiederhold N.P. (2019). In Vitro Activity of Essential Oils against Gram-Positive and Gram-Negative Clinical Isolates, Including Carbapenem-Resistant Enterobacteriaceae. Open Forum Infect. Dis..

[44] Oliva A., Costantini S., De Angelis M., Garzoli S., Božović M., Mascellino M.T., Vullo V., Ragno R. (2018). High Potency of Melaleuca Alternifolia Essential Oil against Multi-Drug Resistant Gram-Negative Bacteria and Methicillin-Resistant *Staphylococcus aureus*. Molecules.

[45] Qian W., Liu M., Fu Y., Wang T., Zhang J., Yang M., Sun Z., Li X., Li Y. (2020). Antimicrobial and Antibiofilm Activities of Citral against Carbapenem-Resistant Enterobacter Cloacae. Foodborne Pathog. Dis..

[46] Ginting E.V., Retnaningrum E., Widiasih D.A. (2021). Antibacterial Activity of Clove (*Syzygium aromaticum*) and Cinnamon (*Cinnamomum burmannii*) Essential Oil against Extended-Spectrum β-Lactamase-Producing Bacteria. Vet. World.

[47] Sharifi-Rad J., Mnayer D., Roointan A., Shahri F., Ayatollahi S.A.M., Sharifi-Rad M., Molaee N. (2016). Antibacterial Activities of Essential Oils from Iranian Medicinal Plants on Extended-Spectrum Î^2^-Lactamase-Producing *Escherichia coli*. Cell. Mol. Biol..

[48] Kwiatkowski P., Pruss A., Grygorcewicz B., Wojciuk B., Dołȩgowska B., Giedrys-Kalemba S., Kochan E., Sienkiewicz M. (2018). Preliminary Study on the Antibacterial Activity of Essential Oils Alone and in Combination with Gentamicin against Extended-Spectrum β-Lactamase-Producing and New Delhi Metallo-β-Lactamase-1-Producing *Klebsiella pneumoniae* Isolates. Microb. Drug Resist..

[49] Benameur Q., Gervasi T., Pellizzeri V., Pľuchtová M., Tali-Maama H., Assaous F., Guettou B., Rahal K., Gruľová D., Dugo G. (2019). Antibacterial Activity of Thymus Vulgaris Essential Oil Alone and in Combination with Cefotaxime against BlaESBL Producing Multidrug Resistant Enterobacteriaceae Isolates. Nat. Prod. Res..

[50] Aouadhi C., Jouini A., Mechichi D., Boulares M., Hamrouni S. (2022). Characterization of Primary Action Mode of Eight Essential Oils and Evaluation of Their Antibacterial Effect Against Extended-Spectrum β-Lactamase (ESBL)-Producing *Escherichia coli* Inoculated in Turkey Meat. Molecules.

[51] Saliu E.-M., Ren H., Goodarzi Boroojeni F., Zentek J., Vahjen W. (2020). The Impact of Direct-Fed Microbials and Phytogenic Feed Additives on Prevalence and Transfer of Extended-Spectrum Beta-Lactamase Genes in Broiler Chicken. Microorganisms.

[52] Motola G., Hafez H.M., Brüggemann-Schwarze S. (2020). Efficacy of Six Disinfection Methods against Extended-Spectrum Beta-Lactamase (ESBL) Producing *E. coli* on Eggshells in Vitro. PLoS ONE.

[53] Krishnamoorthy R., Athinarayanan J., Periasamy V.S., Adisa A.R., Al-Shuniaber M.A., Gassem M.A., Alshatwi A.A. (2018). Antimicrobial Activity of Nanoemulsion on Drug-Resistant Bacterial Pathogens. Microb. Pathog..

[54] Tebrün W., Motola G., Hafez M.H., Bachmeier J., Schmidt V., Renfert K., Reichelt C., Brüggemann-Schwarze S., Pees M. (2020). Preliminary Study: Health and Performance Assessment in Broiler Chicks Following Application of Six Different Hatching Egg Disinfection Protocols. PLoS ONE.

[55] Shrivastav A., Sharma R.K., Shrivastava N., Gautam V., Jain S.K. (2019). Study of Inhibitory Potential and Percent Inhibition of Oil of *Syzygium aromaticum* and Leaves of *Ocimum sanctum* on ESBL Enzyme from *Escherichia Coli* in Broilers of Jabalpur. Indian J. Pharmacol..

[56] Kaskatepe B., Yildiz S.S., Kiymaci M.E., Yazgan A.N., Cesur S., Erdem S.A. (2017). Chemical Composition and Antimicrobial Activity of the Commercial *Origanum onites* L. Oil against Nosocomial Carbapenem Resistant Extended Spectrum Beta Lactamase Producer *Escherichia coli* Isolates. Acta Biol. Hung..

[57] Tadić V., Oliva A., Božović M., Cipolla A., De Angelis M., Vullo V., Garzoli S., Ragno R. (2017). Chemical and Antimicrobial Analyses of *Sideritis romana* L. Subsp. *Purpurea* (Tal. Ex Benth.) Heywood, an Endemic of the Western Balkan. Molecules.

[58] Iseppi R., Di Cerbo A., Aloisi P., Manelli M., Pellesi V., Provenzano C., Camellini S., Messi P., Sabia C. (2020). In Vitro Activity of Essential Oils against Planktonic and Biofilm Cells of Extended-Spectrum β-Lactamase (ESBL)/Carbapenamase-Producing Gram-Negative Bacteria Involved in Human Nosocomial Infections. Antibiotics.

[59] Gāliņa D., Radenkovs V., Kviesis J., Valdovska A. (2022). Effect of Essential Oils Supplemented with Caprylic Acid and Sodium Chloride against Faecal ESBL-Producing *Escherichia coli* Isolated from Pigs. Antibiotics.

[60] Benameur Q., Gervasi T., Pellizzeri V., Pľuchtová M., Gruľová D., Cicero N., Meriem-Hind B. (2021). Comparison of Sensitivity to a Commercial Origanum Vulgare Essential Oil between Extended-Spectrum β-Lactamases (ESBL-) and Non-ESBL-Producing Enterobacteriaceae Isolates. Nat. Prod. Res..

[61] Contreras-Moreno B.Z., Velasco J.J., Rojas J.D.C., Méndez L.D.C., Celis M.T. (2016). Antimicrobial Activity of Essential Oil of *Pimenta racemosa* Var. *Racemosa* (Myrtaceae) Leaves. J. Pharm. Pharmacogn. Res..

[62] de Souza G.H.A., dos Santos Radai J.A., Vaz M.S.M., da Silva K.E., Fraga T.L., Barbosa L.S., Simionatto S. (2021). In Vitro and In Vivo Antibacterial Activity Assays of Carvacrol: A Candidate for Development of Innovative Treatments against KPC-Producing *Klebsiella pneumoniae*. PLoS ONE.

[63] Dhara L., Tripathi A. (2020). Sub-Acute Toxicological and Behavioural Effects of Two Candidate Therapeutics, Cinnamaldehyde and Eugenol, for Treatment of ESBL Producing-Quinolone Resistant Pathogenic Enterobacteriaceae. Clin. Exp. Pharmacol. Physiol..

[64] Dhara L., Tripathi A. (2020). Cinnamaldehyde: A Compound with Antimicrobial and Synergistic Activity against ESBL-Producing Quinolone-Resistant Pathogenic Enterobacteriaceae. Eur. J. Clin. Microbiol. Infect. Dis..

[65] Gore M.R., Raut D., Aruna K. (2021). Antimicrobial activity of *Pimenta dioica* (L.) merr. leaves and its synergistic activity with ampicillin against esbl producing clinical isolates. J. Microbiol. Biotechnol. Food Sci..

[66] Khan I., Bahuguna A., Kumar P., Bajpai V.K., Kang S.C. (2017). Antimicrobial Potential of Carvacrol against Uropathogenic *Escherichia coli* via Membrane Disruption, Depolarization, and Reactive Oxygen Species Generation. Front. Microbiol..

[67] Köse E.O. (2022). In Vitro Activity of Carvacrol in Combination with Meropenem against Carbapenem-Resistant *Klebsiella pneumoniae*. Folia Microbiol..

[68] Qian W., Sun Z., Wang T., Yang M., Liu M., Zhang J., Li Y. (2020). Antimicrobial Activity of Eugenol against Carbapenem-Resistant *Klebsiella pneumoniae* and Its Effect on Biofilms. Microb. Pathog..

[69] Ramachandran G., Rajivgandhi G.N., Murugan S., Alharbi N.S., Kadaikunnan S., Khaled J.M., Almanaa T.N., Manoharan N., Li W.J. (2020). Anti-Carbapenamase Activity of Camellia Japonica Essential Oil against Isolated Carbapenem Resistant *Klebsiella pneumoniae* (MN396685). Saudi J. Biol. Sci..

[70] Sara M., Yamina B., Ramazan E., Mesut G., Selma A. (2021). Dietary Risk of Bla ESBL Producing Multidrug Resistant Enterobacteriaceae and Their Inhibition by Artemisia Herba-Alba and Thymus Algeriensis Essential Oils. J. Essent. Oil Bear. Plants.

[71] Vazquez N.M., Mariani F., Torres P.S., Moreno S., Galvan E.M. (2020). Cell Death and Biomass Reduction in Biofilms of Multidrug Resistant Extended Spectrum β-Lactamase-Producing Uropathogenic *Escherichia coli* Isolates by 1,8-Cineole. PLoS ONE.

[72] Vázquez-López R., Solano-Gálvez S.G., Vignon-Whaley J.J.J., Vaamonde J.A.A., Alonzo L.A.P., Reséndiz A.R., Álvarez M.M., López E.N.V., Franyuti-Kelly G., Álvarez-Hernández D.A. (2020). *Acinetobacter baumannii* Resistance: A Real Challenge for Clinicians. Antibiotics.

[73] Lee C.R., Lee J.H., Park M., Park K.S., Bae I.K., Kim Y.B., Cha C.J., Jeong B.C., Lee S.H. (2017). Biology of *Acinetobacter baumannii*: Pathogenesis, Antibiotic Resistance Mechanisms, and Prospective Treatment Options. Front. Cell. Infect. Microbiol..

[74] Ramirez M.S., Bonomo R.A., Tolmasky M.E. (2020). Carbapenemases: Transforming *Acinetobacter baumannii* into a yet More Dangerous Menace. Biomolecules.

[75] Kyriakidis I., Vasileiou E., Pana Z.D., Tragiannidis A. (2021). *Acinetobacter baumannii* Antibiotic Resistance Mechanisms. Pathogens.

[76] Cai Y., Chai D., Wang R., Liang B., Bai N. (2012). Colistin Resistance of *Acinetobacter baumannii*: Clinical Reports, Mechanisms and Antimicrobial Strategies. J. Antimicrob. Chemother..

[77] Ibrahim S., Al-Saryi N., Al-Kadmy I.M.S., Aziz S.N. (2021). Multidrug-Resistant *Acinetobacter baumannii* as an Emerging Concern in Hospitals. Mol. Biol. Rep..

[78] Yang C.H., Su P.W., Moi S.H., Chuang L.Y. (2019). Biofilm Formation in *Acinetobacter baumannii*: Genotype-Phenotype Correlation. Molecules.

[79] Gómara M., Ramón-García S. (2019). The FICI Paradigm: Correcting Flaws in Antimicrobial in vitro Synergy Screens at Their Inception. Biochem. Pharmacol..

[80] Pang Z., Raudonis R., Glick B.R., Lin T.J., Cheng Z. (2019). Antibiotic Resistance in *Pseudomonas aeruginosa*: Mechanisms and Alternative Therapeutic Strategies. Biotechnol. Adv..

[81] Stover C.K., Pham X.Q., Erwin A.L., Mizoguchi S.D., Warrener P., Hickey M.J., Brinkman F.S.L., Hufnagle W.O., Kowallk D.J., Lagrou M. (2000). Complete Genome Sequence of *Pseudomonas aeruginosa* PAO1, an Opportunistic Pathogen. Nature.

[82] Thi M.T.T., Wibowo D., Rehm B.H.A. (2020). *Pseudomonas aeruginosa* Biofilms. Int. J. Mol. Sci..

[83] Botelho J., Grosso F., Peixe L. (2019). Antibiotic Resistance in *Pseudomonas aeruginosa*–Mechanisms, Epidemiology and Evolution. Drug Resist. Updat..

[84] Hancock R.E.W., Brinkman F.S.L. (2002). Function of *Pseudomonas Porins* in Uptake and Efflux. Annu. Rev. Microbiol..

[85] Dreier J., Ruggerone P. (2015). Interaction of Antibacterial Compounds with RND Efflux Pumps in *Pseudomonas aeruginosa*. Front. Microbiol..

[86] Langendonk R.F., Neill D.R., Fothergill J.L. (2021). The Building Blocks of Antimicrobial Resistance in *Pseudomonas aeruginosa*: Implications for Current Resistance-Breaking Therapies. Front. Cell. Infect. Microbiol..

[87] Podschun R., Ullmann U. (1998). *Klebsiella* Spp. as Nosocomial Pathogens. Clin. Microbiol. Rev..

[88] Zhu J., Wang T., Chen L., Du H. (2021). Virulence Factors in Hypervirulent *Klebsiella pneumoniae*. Front. Microbiol..

[89] Lam M.M.C., Wick R.R., Watts S.C., Cerdeira L.T., Wyres K.L., Holt K.E. (2021). A Genomic Surveillance Framework and Genotyping Tool for *Klebsiella pneumoniae* and Its Related Species Complex. Nat. Commun..

[90] Martin R.M., Bachman M.A. (2018). Colonization, Infection, and the Accessory Genome of *Klebsiella pneumoniae*. Front. Cell. Infect. Microbiol..

[91] Shon A.S., Bajwa R.P.S., Russo T.A. (2013). Hypervirulent (Hypermucoviscous) *Klebsiella pneumoniae*: A New and Dangerous Breed. Virulence.

[92] Wang G., Zhao G., Chao X., Xie L., Wang H. (2020). The Characteristic of Virulence, Biofilm and Antibiotic Resistance of *Klebsiella pneumoniae*. Int. J. Environ. Res. Public Health.

[93] Gomez-Simmonds A., Uhlemann A.C. (2017). Clinical Implications of Genomic Adaptation and Evolution of Carbapenem-Resistant *Klebsiella pneumoniae*. J. Infect. Dis..

[94] Llobet E., Martínez-Moliner V., Moranta D., Dahlström K.M., Regueiro V., Tomása A., Cano V., Pérez-Gutiérrez C., Frank C.G., Fernández-Carrasco H. (2015). Deciphering Tissue-Induced *Klebsiella pneumoniae* Lipid a Structure. Proc. Natl. Acad. Sci. USA.

[95] Paczosa M.K., Mecsas J. (2016). Klebsiella Pneumoniae: Going on the Offense with a Strong Defense. Microbiol. Mol. Biol. Rev..

[96] Nirwati H., Sinanjung K., Fahrunissa F., Wijaya F., Napitupulu S., Hati V.P., Hakim M.S., Meliala A., Aman A.T., Nuryastuti T. (2019). Biofilm Formation and Antibiotic Resistance of *Klebsiella pneumoniae* Isolated from Clinical Samples in a Tertiary Care Hospital, Klaten, Indonesia. BMC Proc..

[97] Wyres K.L., Holt K.E. (2018). *Klebsiella pneumoniae* as a Key Trafficker of Drug Resistance Genes from Environmental to Clinically Important Bacteria. Curr. Opin. Microbiol..

[98] Srinivasan V.B., Venkataramaiah M., Mondal A., Vaidyanathan V., Govil T., Rajamohan G. (2012). Functional Characterization of a Novel Outer Membrane Porin KpnO, Regulated by PhoBR Two-Component System in *Klebsiella pneumoniae* NTUH-K2044. PLoS ONE.

[99] Peirano G., Ahmed-Bentley J., Fuller J., Rubin J.E., Pitout J.D.D. (2014). Travel-Related Carbapenemase-Producing Gram-Negative Bacteria in Alberta, Canada: The First 3 Years. J. Clin. Microbiol..

[100] Mazzariol A., Zuliani J., Cornaglia G., Rossolini G.M., Fontana R. (2002). AcrAB Efflux System: Expression and Contribution to Fluoroquinolone Resistance in *Klebsiella* spp.. Antimicrob. Agents Chemother..

[101] Ping Y., Ogawa W., Kuroda T., Tsuchiya T. (2007). Gene Cloning and Characterization of KdeA, a Multidrug Efflux Pump from *Klebsiella pneumoniae*. Biol. Pharm. Bull..

[102] Wong M.H.Y., Chan E.W.C., Chen S. (2015). Evolution and Dissemination of OqxAB-like Efflux Pumps, an Emerging Quinolone Resistance Determinant among Members of Enterobacteriaceae. Antimicrob. Agents Chemother..

[103] Martínez-Martínez L., Hernández-Allés S., Albertí S., Tomás J.M., Benedi V.J., Jacoby G.A. (1996). In Vivo Selection of Porin-Deficient Mutants of *Klebsiella pneumoniae* with Increased Resistance to Cefoxitin and Expanded-Spectrum Cephalosporins. Antimicrob. Agents Chemother..

[104] Nam Y.S., Cho S.Y., Yang H.Y., Park K.S., Jang J.H., Kim Y.T., Jeong J.W., Suh J.T., Lee H.J. (2013). Investigation of Mutation Distribution in DNA Gyrase and Topoisomerase IV Genes in Ciprofloxacin-Non-Susceptible Enterobacteriaceae Isolated from Blood Cultures in a Tertiary Care University Hospital in South Korea, 2005–2010. Int. J. Antimicrob. Agents.

[105] Clements A., Tull D., Jenney A.W., Farn J.L., Kim S.H., Bishop R.E., McPhee J.B., Hancock R.E.W., Hartland E.L., Pearse M.J. (2007). Secondary Acylation of *Klebsiella pneumoniae* Lipopolysaccharide Contributes to Sensitivity to Antibacterial Peptides. J. Biol. Chem..

[106] Navon-Venezia S., Kondratyeva K., Carattoli A. (2017). Klebsiella Pneumoniae: A Major Worldwide Source and Shuttle for Antibiotic Resistance. FEMS Microbiol. Rev..

[107] Wyres K.L., Lam M.M.C., Holt K.E. (2020). Population Genomics of *Klebsiella pneumoniae*. Nat. Rev. Microbiol..

[108] David S., Reuter S., Harris S.R., Glasner C., Feltwell T., Argimon S., Abudahab K., Goater R., Giani T., Errico G. (2019). Epidemic of Carbapenem-Resistant *Klebsiella pneumoniae* in Europe Is Driven by Nosocomial Spread. Nat. Microbiol..

[109] Reyes J., Aguilar A.C., Caicedo A. (2019). Carbapenem-Resistant *Klebsiella pneumoniae*: Microbiology Key Points for Clinical Practice. Int. J. Gen. Med..

[110] Xu L., Sun X., Ma X. (2017). Systematic Review and Meta-Analysis of Mortality of Patients Infected with Carbapenem-Resistant *Klebsiella pneumoniae*. Ann. Clin. Microbiol. Antimicrob..

[111] Ernst C.M., Braxton J.R., Rodriguez-Osorio C.A., Zagieboylo A.P., Li L., Pironti A., Manson A.L., Nair A.V., Benson M., Cummins K. (2020). Adaptive Evolution of Virulence and Persistence in Carbapenem-Resistant *Klebsiella pneumoniae*. Nat. Med..

[112] Allocati N., Masulli M., Alexeyev M.F., Di Ilio C. (2013). *Escherichia coli* in Europe: An Overview. Int. J. Environ. Res. Public Health.

[113] Denamur E., Clermont O., Bonacorsi S., Gordon D. (2021). The Population Genetics of Pathogenic *Escherichia coli*. Nat. Rev. Microbiol..

[114] Lee S.Y. (2009). Systems Biology and Biotechnology of Escherichia coli.

[115] Liu B., Furevi A., Perepelov A.V., Guo X., Cao H., Wang Q., Reeves P.R., Knirel Y.A., Wang L., Widmalm G. (2020). Structure and Genetics of *Escherichia coli* O Antigens. FEMS Microbiol. Rev..

[116] Sharma G., Sharma S., Sharma P., Chandola D., Dang S., Gupta S., Gabrani R. (2016). *Escherichia Coli* Biofilm: Development and Therapeutic Strategies. J. Appl. Microbiol..

[117] Choi U., Lee C.R. (2019). Distinct Roles of Outer Membrane Porins in Antibiotic Resistance and Membrane Integrity in *Escherichia coli*. Front. Microbiol..

[118] Poirel L., Madec J.-Y., Lupo A., Schink A.-K., Kieffer N., Nordmann P., Schwarz S. (2018). Antimicrobial Resistance in *Escherichia coli*. Microbiol. Spectr..

[119] Jacoby G.A. (2009). AmpC Β-Lactamases. Clin. Microbiol. Rev..

[120] Dagher C., Salloum T., Alousi S., Arabaghian H., Araj G.F., Tokajian S. (2018). Molecular Characterization of Carbapenem Resistant *Escherichia coli* Recovered from a Tertiary Hospital in Lebanon. PLoS ONE.

[121] Tian X., Zheng X., Sun Y., Fang R., Zhang S., Zhang X., Lin J., Cao J., Zhou T. (2020). Molecular Mechanisms and Epidemiology of Carbapenem-Resistant *Escherichia coli* Isolated from Chinese Patients during 2002–2017. Infect. Drug Resist..

[122] Gurung S., Kafle S., Dhungel B., Adhikari N., Shrestha U.T., Adhikari B., Banjara M.R., Rijal K.R., Ghimire P. (2020). Detection of Oxa-48 Gene in Carbapenem-Resistant *Escherichia coli* and *Klebsiella pneumoniae* from Urine Samples. Infect. Drug Resist..

[123] Lombrea A., Antal D., Ardelean F., Avram S., Pavel I.Z., Vlaia L., Mut A.M., Diaconeasa Z., Dehelean C.A., Soica C. (2020). A Recent Insight Regarding the Phytochemistry and Bioactivity of *Origanum vulgare* L. Essential Oil. Int. J. Mol. Sci..

[124] Ojeda-Sana A.M., van Baren C.M., Elechosa M.A., Juárez M.A., Moreno S. (2013). New Insights into Antibacterial and Antioxidant Activities of Rosemary Essential Oils and Their Main Components. Food Control.

[125] Page M.J., McKenzie J.E., Bossuyt P.M., Boutron I., Hoffmann T.C., Mulrow C.D., Shamseer L., Tetzlaff J.M., Akl E.A., Brennan S.E. (2021). The PRISMA 2020 Statement: An Updated Guideline for Reporting Systematic Reviews. Syst. Rev..

